# Plant species, inundation, and sediment grain size control the development of sediment stability in tidal marshes

**DOI:** 10.1002/eap.3078

**Published:** 2025-01-20

**Authors:** Marte M. Stoorvogel, Jaco C. de Smit, Lauren E. Wiesebron, Jim van Belzen, Johan van de Koppel, Stijn Temmerman, Tjeerd J. Bouma

**Affiliations:** ^1^ Department of Estuarine and Delta Systems NIOZ Royal Netherlands Institute for Sea Research Yerseke The Netherlands; ^2^ Department of Physical Geography Utrecht University Utrecht The Netherlands; ^3^ Building with Nature Research Group, Department of Technology, Water & Environment HZ University of Applied Sciences Middelburg The Netherlands; ^4^ Wageningen Marine Research Wageningen University & Research Yerseke The Netherlands; ^5^ Conservation Ecology Group, Groningen Institute for Evolutionary Life Sciences University of Groningen Groningen The Netherlands; ^6^ ECOSPHERE Research Group, Department of Biology University of Antwerp Antwerp Belgium

**Keywords:** inundation duration, nature‐based shoreline protection, pioneer vegetation, sediment erosion resistance, sediment water content, tidal marsh stability

## Abstract

Tidal marshes can contribute to nature‐based shoreline protection by reducing the wave load onto the shore and reducing the erosion of the sediment bed. To implement such nature‐based shoreline erosion protection requires the ability to quickly restore or create highly stable and erosion‐resistant tidal marshes at places where they currently do not yet occur. Therefore, we aim to identify the drivers controlling the rate by which sediment stability builds up in young pioneer marshes. Sediment stability proxies were measured over age gradients spanning 18 years in six tidal marsh sites in the Western Scheldt estuary (SW Netherlands): Three were dominated by *Spartina anglica*, a densely growing pioneer species, and three by *Scirpus maritimus*, a less densely growing pioneer species. Our results showed that the presence of densely growing *Spartina anglica* increased sediment shear strength compared to the unvegetated tidal flat, while less densely growing *Scirpus maritimus* did not. This difference may be related to the contrasting clonal expansion strategies and related root densities of these two pioneer species. Sediment stability did not increase further beyond 6 years of coverage by *Spartina anglica*, implying that the observed effect of *Spartina anglica* on sediment stability occurs fast (<6 years). Furthermore, sediment stability often increased with decreasing inundation duration and sediment water content. This study shows that in order to create erosion‐resistant sediment beds in future marsh restoration projects, the aim should be to create densely vegetated tidal marshes with well‐draining, cohesive sediments at relatively high intertidal elevation. Although the development of erosion resistance takes time, our study demonstrates that in the case of densely growing *Spartina anglica* marshes, increased sediment bed stability can already be reached after 6 years. The ability of *Spartina anglica* marshes to increase sediment bed stability within 6 years, in combination with wave attenuation and sediment accretion, offers promising perspectives to implement marsh restoration projects as a nature‐based shoreline protection strategy that can start to deliver its protective service within a reasonable amount of time.

## INTRODUCTION

Climate change and associated sea‐level rise and increase in storm frequency and intensity (IPCC, 2023; Knutson et al., 2010) lead to elevated flood risks in delta regions. Around the world, many people live along coastlines and this number will only increase in the future (Syvitski et al., 2009), resulting in large social and economic impacts if a flood occurs. The current solution of heightening and strengthening engineered flood defense structures, such as dikes or levees, is expensive and challenging to sustain since it requires much regular maintenance (Temmerman et al., 2013; Tessler et al., 2015). Nature‐based shoreline protection has been suggested as a robust, cost‐effective, and sustainable additional strategy that can increase the protective capacity of engineered flood defenses (Temmerman et al., 2023; van Zelst et al., 2021; Vuik et al., 2019; Zhu, Vuik, et al., 2020). This combination of nature‐based and engineered flood defense strategies is often referred to as a hybrid approach, where hard engineering becomes more efficient by combining it with conservation or creation of ecosystems, such as tidal marshes in front of an engineered dike (Schoonees et al., 2019).

The presence of a tidal marsh with an erosion‐resistant sediment bed in front of a dike contributes to shoreline protection in three ways. First, tidal marshes are effective in dissipating wave energy, thereby attenuating wave loading onto the dike and reducing the risk that a dike breaches during a storm (Zhu, Vuik, et al., 2020), provided that the marsh is wide enough (Möller et al., 2014; Vuik et al., 2016; Willemsen et al., 2020). Having wide marshes requires them to be strong enough to resist lateral erosion (Bouma et al., 2014). Wave attenuation capacity of marsh vegetation increases with increasing vegetation stiffness, frontal area, and plant height and is therefore species‐dependent (Paul et al., 2016; Schoutens et al., 2020; Temmerman et al., 2023; Vuik et al., 2016). Second, if a dike does fail, the presence of an erosion‐resistant tidal marsh fronting that dike reduces the dike breach growth, thereby lowering discharges through the dike breach and ultimately reducing flood damage and casualties in the low‐lying land behind the dike (Zhu, Vuik, et al., 2020). This mechanism only works if the marsh sediment beds are erosion resistant enough to remain intact under the high flow velocities that occur toward the dike breach (Schoutens et al., 2022; Zhu, Vuik, et al., 2020). Thus, having a wide and erosion‐resistant tidal marsh in front of a dike makes coastlines better protected against flooding. Third, tidal marshes can increase their bed surface elevation through sediment accretion (Fagherazzi et al., 2012; Mudd et al., 2010) and biomass growth (Blum et al., 2021), which allows them to grow vertically with sea‐level rise if sediment supply and sedimentation rates are sufficient (Schuerch et al., 2018). Bass et al. (2022) showed that vertical accretion increased with both increasing leaf and stem roughness, and Baaij et al. (2021) showed that stem height and branching level were important determinants of sedimentation, which illustrates that vertical marsh accretion depends on the occurring vegetation species. These three benefits are becoming increasingly important given the sea‐level rise and increasing storm frequency and intensity due to climate warming. This raises the question of how long it takes to restore or create vegetated and erosion‐resistant tidal marshes at places where they currently do not yet occur.

An erosion‐resistant sediment bed can form over time through multiple processes. When sediment particles settle on the bed surface, it will initially form a wet, soft, and highly unstable sediment. By self‐weight consolidation, pore water is expelled from the sediment matrix (Barciela‐Rial et al., 2020; Torfs et al., 1996). This compacts and densifies the sediment structure. Consolidation affects sediment structure, increases bulk density, and decreases water content, resulting in an increase in cohesive strength between fine‐grained mud particles. Therefore, consolidation gradually increases erosion resistance over time (Chen et al., 2012; Grabowski et al., 2011; Zhou et al., 2016). Another important process for the development of an erosion‐resistant tidal marsh sediment bed is vegetation growth. Plants can affect sediment stability in several ways. A higher root biomass is associated with higher erosion resistance (Brooks et al., 2021; Chen et al., 2012; Wang et al., 2017), since roots stabilize sediment and reinforce the sediment matrix (Brooks et al., 2021; Grabowski et al., 2011; Gyssels et al., 2005; Lo et al., 2017). Plants can also indirectly increase sediment stability by modifying sediment properties (Feagin et al., 2009). Plants can capture fine, cohesive sediments (Feagin et al., 2015; James et al., 2019) and add organic matter to the sediment bed (Feagin et al., 2015). The increased clay and organic matter content will lead to higher cohesion and in turn enhance sediment stability (Feagin et al., 2015; Joensuu et al., 2018). Furthermore, sediment pore water is taken up and evapotranspirated by plants (Barciela‐Rial et al., 2023), which may further contribute to lowering the sediment water content and increasing the strength of silt‐ and clay‐rich cohesive sediments.

While we know that mature marshes are often highly erosion resistant (Marin‐Diaz et al., 2022; Schoutens et al., 2022; Wang et al., 2017) and we have insight into which factors can drive sediment stability and erosion resistance (e.g., Brooks et al., 2021; Grabowski et al., 2011; Lo et al., 2017), it remains largely unknown at which rates sediment stability and erosion resistance can build up in marsh restoration and creation projects. To implement marsh restoration and creation for coastal safety, it is necessary to gain an in‐depth understanding of the factors and processes that drive the rate of sediment stability development. This will allow us to better predict how sediment stability and erosion resistance will build up over time.

In this study, we therefore examined the rate at which sediment stability builds up under the influence of establishment of pioneer marsh vegetation species on initially bare tidal flats and by which factors this rate is influenced. To study this rate under pioneer species that grow at different densities, we measured proxies of sediment stability over an age gradient in six tidal marshes in the Western Scheldt estuary in the Netherlands. In three of these tidal marshes, densely growing *Spartina anglica* occurs as the dominant pioneer species, whereas less densely growing *Scirpus maritimus* is the dominant pioneer species in the other three tidal marshes. By determining tidal marsh age and inundation duration and by measuring sediment properties, we were able to study the effects of these variables on the buildup of sediment stability.

## METHODS

### Study locations and dominant pioneer tidal marsh species

**FIGURE 1:**
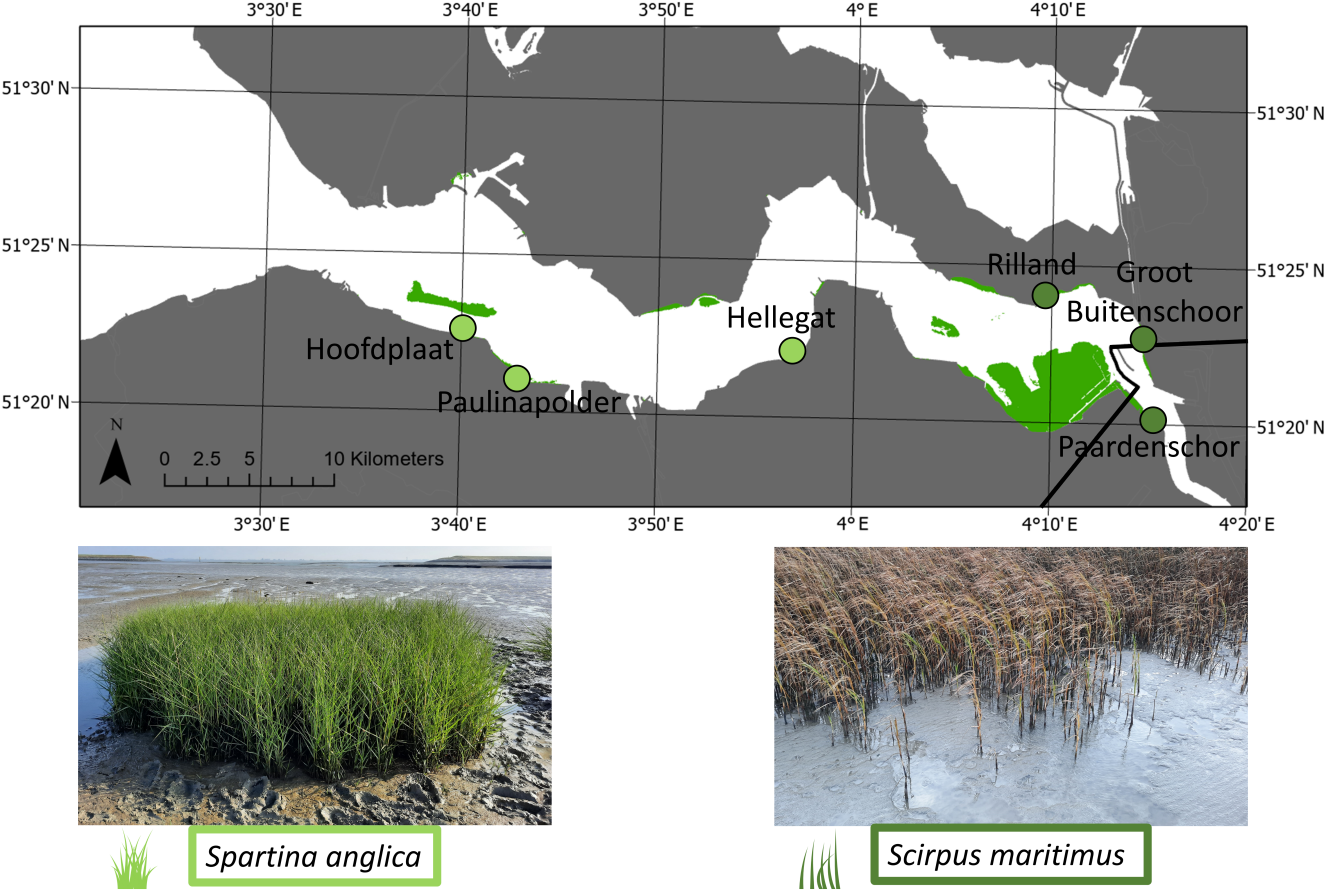
Map of the Western Scheldt estuary indicating the location of the studied tidal marshes of Hoofdplaat, Paulinapolder, Hellegat, Rilland, Groot Buitenschoor, and Paardenschor. The tidal marshes indicated with light green have *Spartina* as dominant pioneer species, the brackish marshes indicated with dark green have *Scirpus* as dominant pioneer species. The extent of tidal marshes in the Western Scheldt is shown in green, and the black line is the Dutch–Belgian border. Photographs of *Spartina anglica* and *Scirpus maritimus* were taken by Marte M. Stoorvogel. Drawings of *Spartina* and *Scirpus* from Creazilla.com under a Public Domain (CC0) license.

To determine the effect of different pioneer vegetation species on the buildup of sediment stability over time, we studied six expanding tidal marshes in the Western Scheldt estuary (southwest of the Netherlands) in 2021 and 2022 (Figure 1). Tidal flat elevation in the Western Scheldt estuary has on average increased with 1.44 cm per year over the last two decades, which is hypothesized to result from navigation channel dredging (Fivash et al., 2023). This increase in elevation has led to increasingly suitable conditions for vegetation growth (Cao, Zhu, van Belzen, et al., 2021), resulting in many expanding tidal marshes in the Western Scheldt estuary, including the ones that were studied here. To compare the effect of plant species, we studied tidal marshes with different dominant pioneer species, which grow at different densities. Densely growing *Spartina anglica* (herein referred to as *Spartina*) was the dominant pioneer species in the studied tidal marshes of Hoofdplaat, Paulinapolder, and Hellegat, which lie further toward the mouth of the estuary close to Terneuzen where average salinity was 24.2‰ between 1996 and 2019 (Deltares, 2021) (Figure 1; Appendix S1: Figure S1). Less densely growing *Scirpus maritimus* (herein referred to as *Scirpus*) was the dominant pioneer species in the studied brackish marshes of Rilland, Groot Buitenschoor, and Paardenschor, which lie further into the estuary close to Schaar van Ouden Doel where average salinity was 9.3‰ between 1996 and 2019 (Deltares, 2021) (Figure 1; Appendix S1: Figure S1). High marsh vegetation was *Elymus repens* (herein referred to as *Elymus*) at the *Spartina* marshes and *Phragmites australis* (herein referred to as *Phragmites*) at the *Scirpus* marshes. *Spartina* and *Elymus* are more salt tolerant than *Scirpus* and *Phragmites* (Zhu, Yang, et al., 2020), which is why these species occur at different locations along the salinity gradient within the estuary. The average suspended particulate matter concentration between 1995 and 2015 of the main channel was 36.9 ± 17.2 mg/L in the zone of the salt *Spartina* marshes and 81 ± 47.5 mg/L in the zone of the brackish *Scirpus* marshes (Cox et al., 2019).

### Defining age gradients within the tidal marshes

**FIGURE 2:**
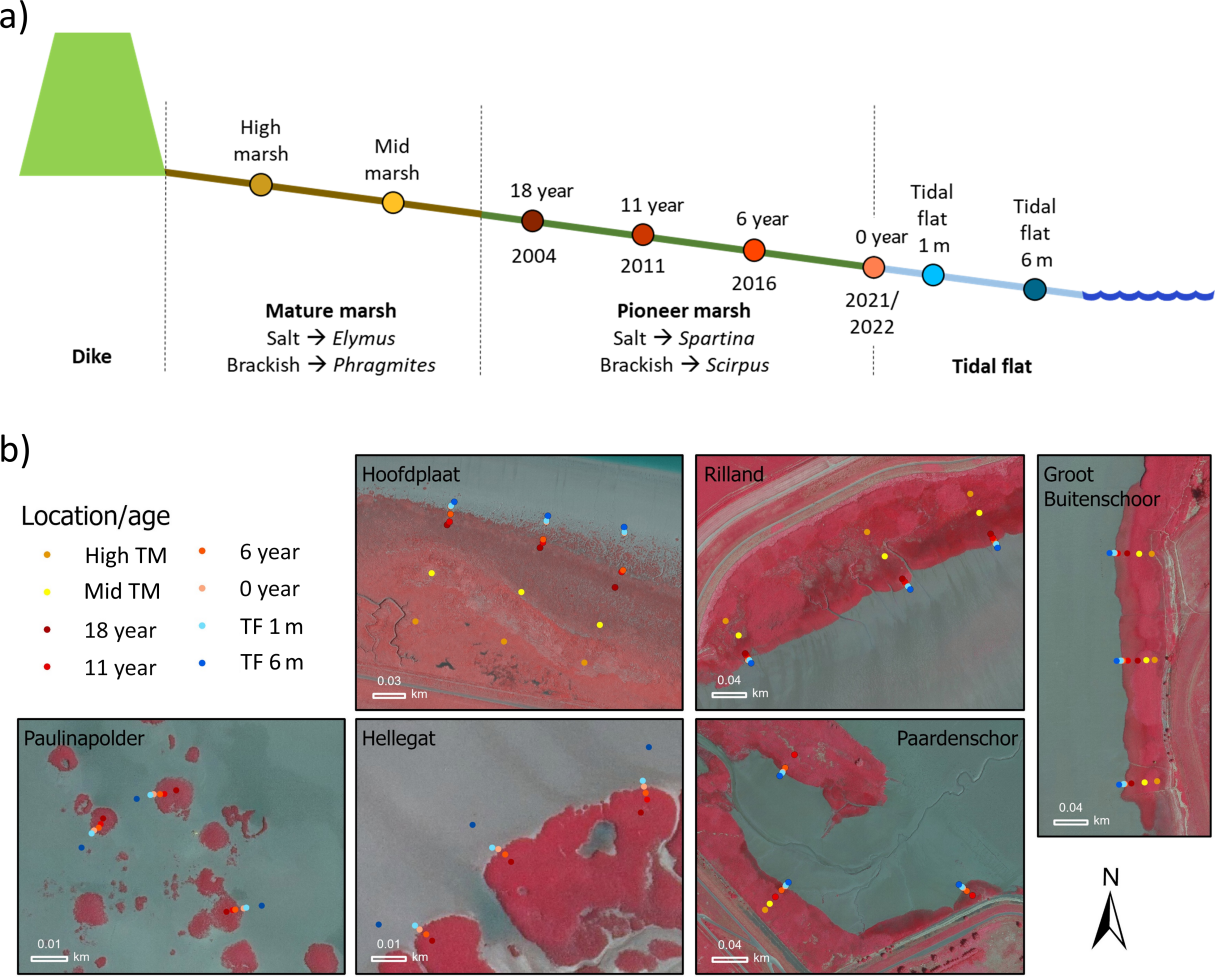
(a) Transects from the mature marsh, over the pioneer zone, to the tidal flat. Dots indicate the measurement locations. These locations are equally spread over the mature marsh, based on historical vegetation edges at the pioneer tidal marsh, and based on distance from the current tidal marsh edge on the tidal flat. (b) Locations of the different measurement locations in the six studied tidal marshes. False color aerial images are of 2022 from Rijkswaterstaat. Locations “High TM” and “Mid TM” are mature tidal marsh, locations “18 year,” “11 year,” and “6 year” are vegetated with pioneer vegetation, location “0 year” lies on the current edge of pioneer vegetation and bare tidal flat, and the tidal flat (TF) locations are bare.

To study the rate with which sediment stability builds up over time, sediment stability measurements were conducted along three transects per tidal marsh from the high and mature marsh, over the pioneer zone, to the tidal flat (Figure 2). At Paulinapolder and Hellegat, the transects ranged only from the pioneer zone of vegetation tussocks to the tidal flat, as no mature marsh existed at these locations (Figure 2). Since all selected marshes and tussocks were expanding, these transects represent time series. The historical tidal marsh vegetation edges of different years (2004, 2011, 2016) were identified by previous real‐time kinematic differential GPS (RTK‐DGPS) measurements of the position of the marsh edges (data from Jim van Belzen [NIOZ] and Silinski et al. [2016], ±1 cm horizontal accuracy) and aerial images of the Western Scheldt, supplied by Rijkswaterstaat (50 cm resolution in 2004 and 25 cm in 2011 and 2016). We measured sediment stability at the *Spartina* and *Scirpus* edges of 2004 (18 years vegetated), 2011 (11 years vegetated), 2016 (6 years vegetated), and the current vegetation edge in 2021 or 2022 (Figure 2). In addition, we took two measurements on the tidal flat 1 and 6 m away from the current vegetation edge and two measurements on the mature marsh equally distributed between the dike and the mature‐pioneer marsh edge (Figure 2; Appendix S2: Table S1). This enabled us to study (1) the effect of vegetation presence on sediment stability and (2) the buildup rate of sediment stability over time under vegetation.

### Field measurements as proxies for erosion resistance

We used an RTK‐DGPS (Trimble, USA, horizontal accuracy ±3 mm, vertical accuracy 5 mm) to find the planned measurement locations in the field. The exact coordinates and elevation (m + normaal amsterdams peil) of the measured locations were recorded using this same RTK‐DGPS. At all measurement locations, we measured sediment properties that can be used as proxies for erosion resistance: sediment shear strength and penetration resistance. Sediment shear strength and penetration resistance have both been shown to relate to how well tidal marsh sediment beds can resist hydrodynamic forces (Amer et al., 2017; Marin‐Diaz et al., 2021; Watts et al., 2003). Surface shear strength was measured with a pocket shear vane tester (Eijkelkamp, the Netherlands). The shear strength of deeper sediment layers was determined with a field inspection shear vane tester (Eijkelkamp, the Netherlands) at 10, 20, 30, and 40 cm. If the pocket shear vane tester and field inspection shear vane tester were turned a full revolution without any sediment movement, a minimum shear strength was noted down. This was 3.2 kPa for surface shear strength and 65 kPa for the deeper layers, which for both instruments is the value corresponding to one full revolution. We often reached the maximum measurable value of 3.2 kPa for surface shear strength at the tidal marsh locations. To measure penetration resistance of the sediment, we used a penetrologger (Eijkelkamp, the Netherlands), until a depth of 40 cm with a 1‐cm interval. Lastly, we collected sediment samples with a gouge auger from 0–10, 10–20, 20–30, and 30–40 cm. Once collected in the field, these sediment samples were put in closed plastic bags to conserve the sediment water content during transport to the laboratory. All measurements and sediment sampling were performed in triplicate.

### Laboratory measurements on sediment properties

All sediment samples were weighed in the laboratory, frozen, and freeze‐dried. Then, they were reweighed to calculate sediment water content. From the 1186 sediment samples that we took, a selection of 149 samples was analyzed for grain size distribution due to financial constraints. With the grain size distribution data of these 149 samples, we aimed to derive relationships between water content and D50 and fine fraction content (<63 μm), and subsequently to estimate the grain size properties of the remaining samples. The selection of 149 samples consisted of one sample of the top layer (0–10 cm) for all measurement locations, and of one sample of the deeper layers (10–20, 20–30, and 30–40 cm) for three measurement locations per tidal marsh: the “6 m tidal flat” location of the first transect, the “18 year” location of the second transect, and the “high marsh” location of the third transect. These samples were sieved over a 1‐mm sieve to remove large particles and belowground biomass. Grain size distribution of the samples was measured with a Mastersizer 2000 (Malvern) based on laser diffraction. With the results of these 149 samples, we tested the relationship between water content and both D50 and fine fraction content (<63 μm) with linear regression.

### Data and statistical analyses

To account for differences in tidal range between the studied tidal marshes, we translated elevation data to inundation duration, that is, the percentage of time a given location is submerged (Balke et al., 2016). For this calculation, we downloaded publicly available water level data from Rijkswaterstaat Waterinfo (Rijkswaterstaat, 2022) for the closest tide gauge per tidal marsh. The measurement stations are located at Prosperpolder (for Groot Buitenschoor and Paardenschor), Bath (for Rilland), “Overloop van Hansweert” (for Hellegat), and Terneuzen (for Paulinapolder and Hoofdplaat) (Appendix S1: Figure S1). The selected period was 5 years, from 1 January 2016 to 31 December 2021.

To include time since vegetation establishment (age) as a parameter in our statistical analyses, we extracted the minimum ages of high‐ and mid‐marsh locations from geomorphological maps of Rijkswaterstaat, which show marsh coverage. The oldest available geomorphological map dates from 1936 and parts of the tidal marshes of Rilland and Hoofdplaat were already vegetated in that year. Therefore, we assumed a minimum age of 86 years for these locations, but they might have been vegetated longer than that. We assigned an age of 0 to the current vegetation edge and both tidal flat locations.

All statistical analyses were performed in R, version 4.2.1 (R Core Team, 2022). We used the dplyr package (Wickham et al., 2022) to calculate averages and SDs of replicates and transects. To study differences between the measurement locations along the age gradients, and thereby study the rate of buildup of sediment stability over time, we applied the Kruskal–Wallis and pairwise Wilcoxon tests to the surface shear strength data (not normally distributed due to the often‐occurring maximum measurable value of 3.2 kPa), and the ANOVA and Tukey honestly significant difference (HSD) tests to the deep shear strength, penetration resistance, and water content data (all normally distributed for the separate tidal marshes). To test relationships between sediment stability proxies (surface and deep shear strength and penetration resistance) and explanatory variables (water content, inundation duration, and age), we applied censored and linear regressions. Since shear strength at 10 cm, average penetration resistance of the top 10 cm, and water content in the top 10 cm are log‐normally distributed when combining the data of all marshes, the data of these parameters were log‐transformed before the regressions. We used the censReg Package (Henningsen et al., 2022) to perform censored regressions to test the relationships between surface shear strength and explanatory variables, because we often reached the measurement limit of 3.2 kPa for surface shear strength, resulting in an upper limit of the data.

## RESULTS

### Sediment stability under pioneer species along an age gradient

**FIGURE 3:**
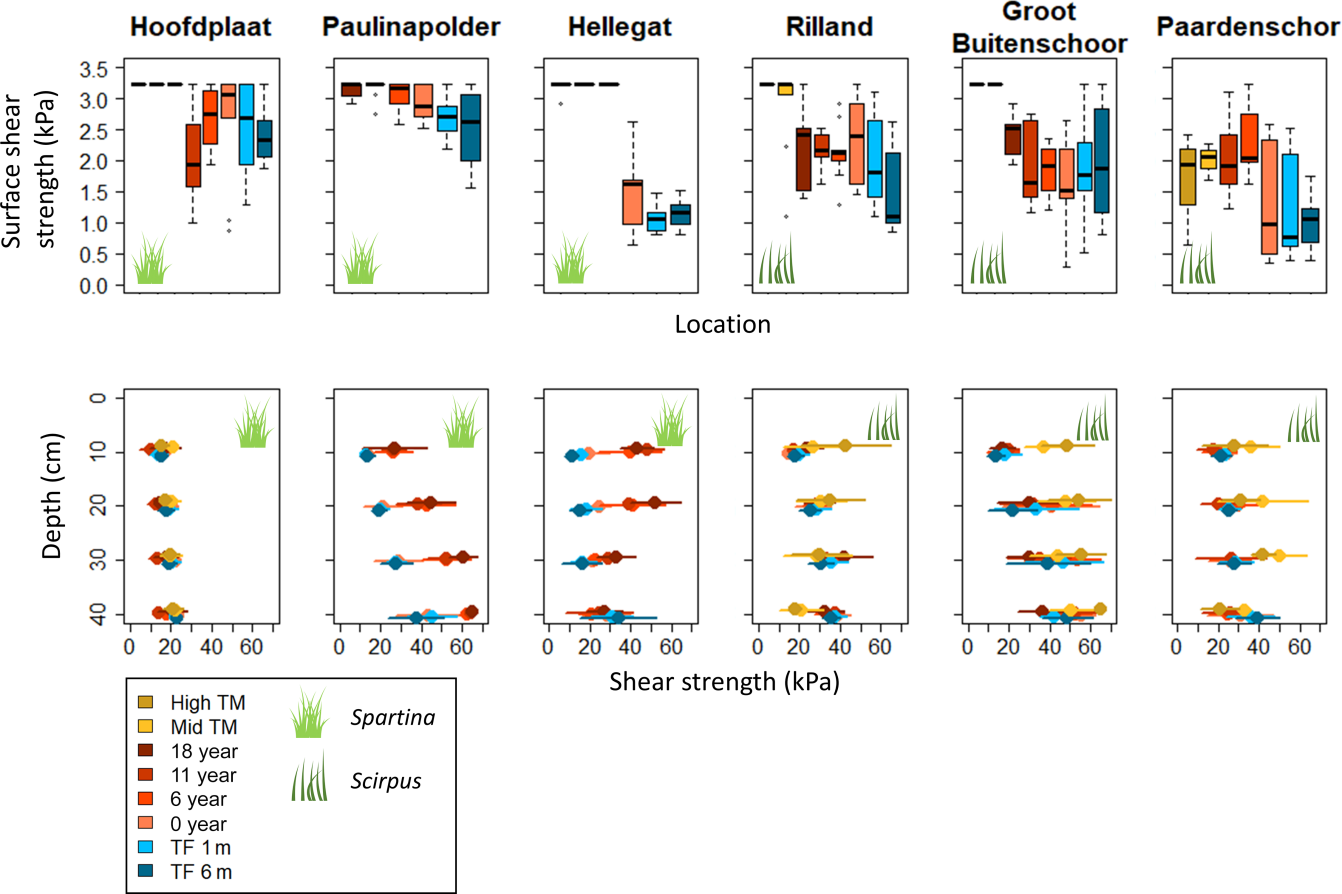
Surface shear strength and shear strength at 10, 20, 30, and 40 cm depth of all measurement locations along the age gradients in the three *Spartina*‐dominated and three *Scirpus*‐dominated tidal marshes. *N* = 9, since each tidal marsh has three transects, and we took three replicates per location. Horizontal lines indicate SDs. Locations “High TM” and “Mid TM” are mature tidal marsh, locations “18 year,” “11 year,” and “6 year” are vegetated with pioneer vegetation, location “0 year” lies on the current edge of pioneer vegetation and bare tidal flat, and the tidal flat (TF) locations are bare. Drawings of *Spartina* and *Scirpus* from Creazilla.com under a Public Domain (CC0) license.

Our results show that *Spartina* and *Scirpus* affect sediment stability differently. In the *Spartina*‐dominated marshes, shear strength was generally greater at vegetated locations than at bare tidal flats. At Paulinapolder and Hellegat, surface shear strength was significantly higher at the locations with *Spartina* than at the current vegetation edge and at the bare tidal flat (Paulinapolder: χ^2^ = 17, *p* < 0.001; Hellegat: χ^2^ = 29, *p* < 0.001; Figure 3). Shear strength was also higher at the *Spartina* locations than at the bare locations in Paulinapolder at 10, 20, 30, and 40 cm depth and in Hellegat at 10, 20, and 30 cm depth (*p* < 0.001 at both marshes and for all aforementioned depths, Figure 3). In contrast, we found no difference in shear strength between pioneer marsh locations and bare locations at the *Scirpus*‐dominated marshes and at the *Spartina*‐dominated marsh of Hoofdplaat, except for a higher surface shear strength in *Scirpus* vegetation than at the bare locations at Paardenschor (χ^2^ = 14, *p* < 0.001). This implies that the presence of *Spartina* generally increases sediment stability, while *Scirpus* does not within 18 years after establishment. Interestingly, shear strength of the surface and of deeper sediment layers did not significantly differ between the *Spartina* vegetation edges of 18, 11, and 6 years old at Paulinapolder (χ^2^ = 2, *p* = 0.362 for surface shear strength) and Hellegat (χ^2^ = 0.67, *p* = 0.717 for surface shear strength; Figure 3). This indicates that shear strength does not further increase between six and 18 years after *Spartina* establishment.

Shear strength at the surface and at depth was frequently significantly higher at the mature marsh locations than at the pioneer marshes. Surface shear strength of the combined mid‐ and high‐marsh locations was significantly higher than of the pioneer marsh locations at Hoofdplaat (*Elymus*), Rilland, and Groot Buitenschoor (both *Phragmites*) (Hoofdplaat: χ^2^ = 13, *p* < 0.001; Rilland: χ^2^ = 22, *p* < 0.001; Groot Buitenschoor: χ^2^ = 34, *p* < 0.001), while this was not the case at Paardenschor (*Phragmites*; χ^2^ = 0.32, *p* = 0.570). Shear strength at 10 cm was higher in the mid and high marsh than at the pioneer marsh at all marshes (Hoofdplaat: *F*_1,43_ = 5, *p* = 0.026; Rilland: *F*_1,43_ = 14, *p* < 0.001; Groot Buitenschoor: *F*_1,43_ = 75, *p* < 0.001; Paardenschor: *F*_1,22_ = 7, *p* = 0.013), and in some cases, this was also true for deeper layers. The maximum value of 3.2 kPa that could be measured with the surface shear vane was often reached in the *Spartina* vegetation and at the high and mid marshes. At all tidal marshes, both the ones dominated by *Spartina* and by *Scirpus*, we found no consistent patterns of shear strength with depth (Figure 3).

**FIGURE 4:**
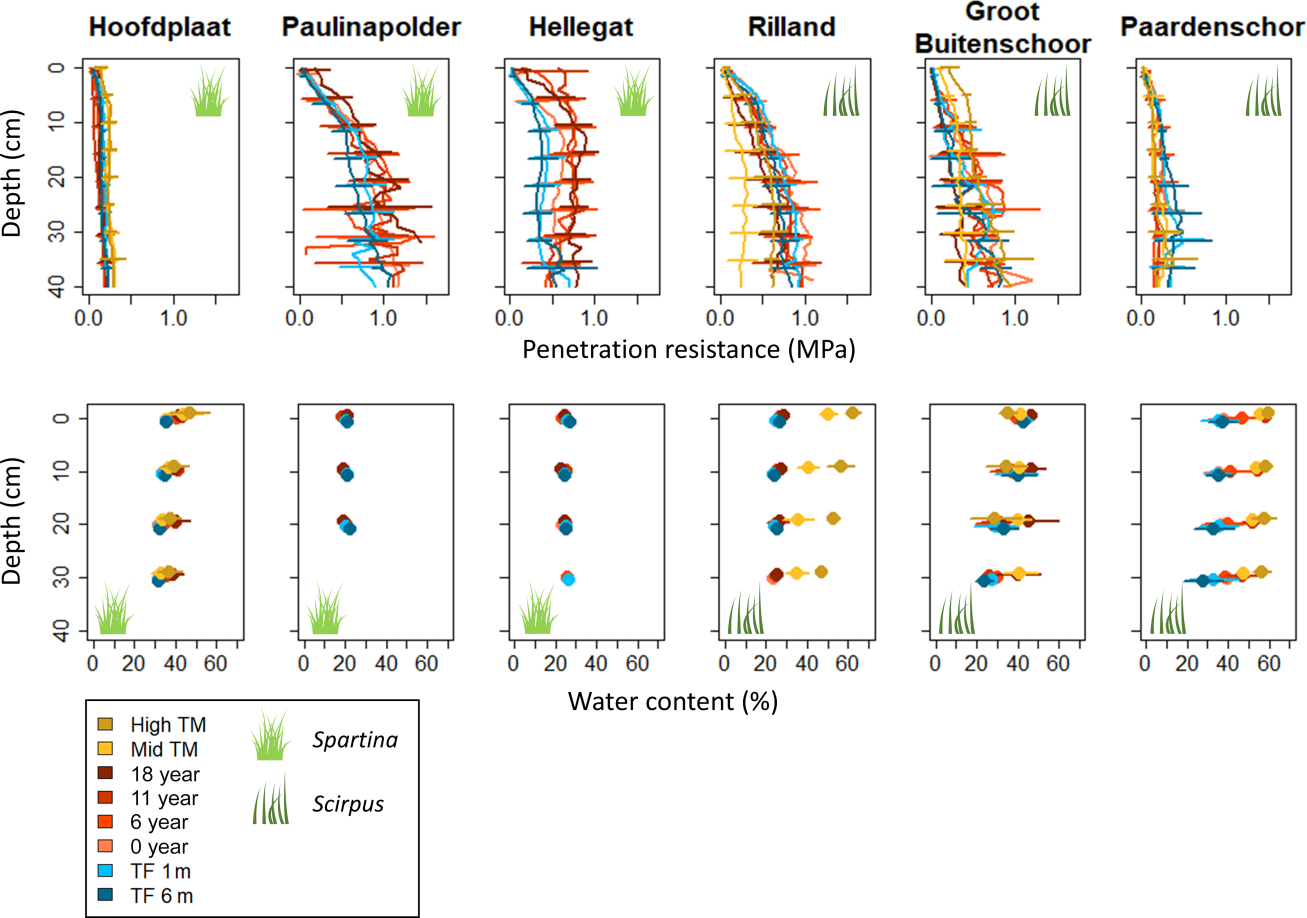
Depth profiles of penetration resistance with a 1‐cm interval and water content from 0–10, 10–20, 20–30, and 30–40 cm depth for all measurement locations along the age gradients in the three *Spartina*‐dominated and three *Scirpus*‐dominated tidal marshes. *N* = 9, since each tidal marsh has three transects, and we took three replicates per location. Horizontal lines indicate SDs. Locations “High TM” and “Mid TM” are mature tidal marsh, locations “18 year,” “11 yea,” and “6 year” are vegetated with pioneer vegetation, location “0 year” lies on the current edge of pioneer vegetation and bare tidal flat, and the tidal flat (TF) locations are bare. Drawings of *Spartina* and *Scirpus* from Creazilla.com under a Public Domain (CC0) license.

We did not find a unified pattern in penetration resistance across marshes and marsh age gradients. At Hellegat, the average penetration resistance until 40 cm depth was significantly higher for the locations with *Spartina* than the current vegetation edge and tidal flat (*F*_1,46_ = 57, *p* < 0.001), which corresponds to the shear strength data (Figures 3 and 4). On the contrary, bare locations had a higher average penetration resistance until 40 cm depth than the *Spartina* pioneer zone at Hoofdplaat (*F*_1,52_ = 10, *p* = 0.002), and than the *Scirpus* pioneer zones at Paardenschor (*F*_1,43_ = 8, *p* = 0.006) and Rilland (*F*_1,52_ = 6, *p* = 0.017). At Paulinapolder (*Spartina* dominated) and Groot Buitenschoor (*Scirpus* dominated), there were no significant penetration resistance differences between bare locations and the pioneer zone (*F*_1,52_ = 0.14, *p* = 0.713, and *F*_1,52_ = 1, *p* = 0.256, respectively). The combined high‐ and mid‐marsh locations at Hoofdplaat (*Elymus*) and Groot Buitenschoor (*Phragmites*) had a higher penetration resistance than the pioneer zone locations (*F*_1,43_ = 51, *p* < 0.001, and *F*_1,42_ = 8, *p* = 0.009, respectively). Penetration resistance at the high and mid marsh at Paardenschor (*Phragmites*) did not differ from that at the *Scirpus* pioneer zone (*F*_1,22_ = 0, *p* = 0.990), while the high and mid marsh at Rilland (*Phragmites*) had a significantly lower penetration resistance than at the pioneer zone (*F*_1,43_ = 40, *p* < 0.001).

Water content differed significantly between tidal marshes (*F*_5,1159_ = 200, *p* < 0.001). Overall, water content was higher at the *Spartina*‐dominated marsh of Hoofdplaat and *Scirpus*‐dominated marshes of Groot Buitenschoor and Paardenschor, and lower at the *Spartina‐*dominated marshes of Paulinapolder and Hellegat and the *Scirpus*‐dominated marsh of Rilland (Figure 4). There were only significant differences in water content at 10 cm depth between the pioneer zone and tidal flat at three marshes. Water content at 10 cm depth was significantly higher in the pioneer zone than at the bare locations at the *Spartina*‐dominated marsh of Hoofdplaat (*F*_1,52_ = 163, *p* < 0.001) and at the *Scirpus*‐dominated marsh of Paardenschor (*F*_1,43_ = 40, *p* < 0.001). In contrast, water content at 10 cm depth at Hellegat was significantly higher at the bare locations than in the *Spartina* pioneer marsh locations (*F*_1,41_ = 20; *p* < 0.001). At the other three marshes, there was no significant difference in water content at 10 cm depth between the pioneer marsh and tidal flat (Paulinapolder: *F*_1,52_ = 2, *p* = 0.125; Rilland: *F*_1,52_ = 20, *p* = 0.243; Groot Buitenschoor: *F*_1,52_ = 0.013, *p* = 0.911). We found a significant difference in water content at 10 cm depth between the combined high and mid marsh and pioneer marsh at two marshes. At Rilland, water content at 10 cm depth was significantly higher in the *Phragmites* high and mid marsh than in the *Scirpus* pioneer marsh (*F*_1,43_ = 409, *p* < 0.001), and at Groot Buitenschoor, this was the opposite (*F*_1,39_ = 7, *p* = 0.010). Water content at 10 cm depth in the high and mid marshes of Hoofdplaat (*Elymus*; *F*_1,43_ = 3, *p* = 0.120) and Paardenschor (*Phragmites*; *F*_1,22_ = 2, *p* = 0.218) did not differ significantly from the pioneer zones.

### Relationships between sediment stability, sediment properties, inundation duration, and marsh age

For the 149 samples that we analyzed for grain size distribution, we studied the relationships between fine fraction content and water content, as well as between D50 and water content (Appendix S3: Figure S1). Both relationships were significant (fine fraction content: *F*_1,147_ = 361, *R* ^2^ = 0.71, *p* < 0.001; D50: *F*_1,147_ = 445, *R* ^2^ = 0.75, *p* < 0.001). A higher fine fraction content corresponds to a higher water content, while a higher D50 leads to a lower water content.

**FIGURE 5:**
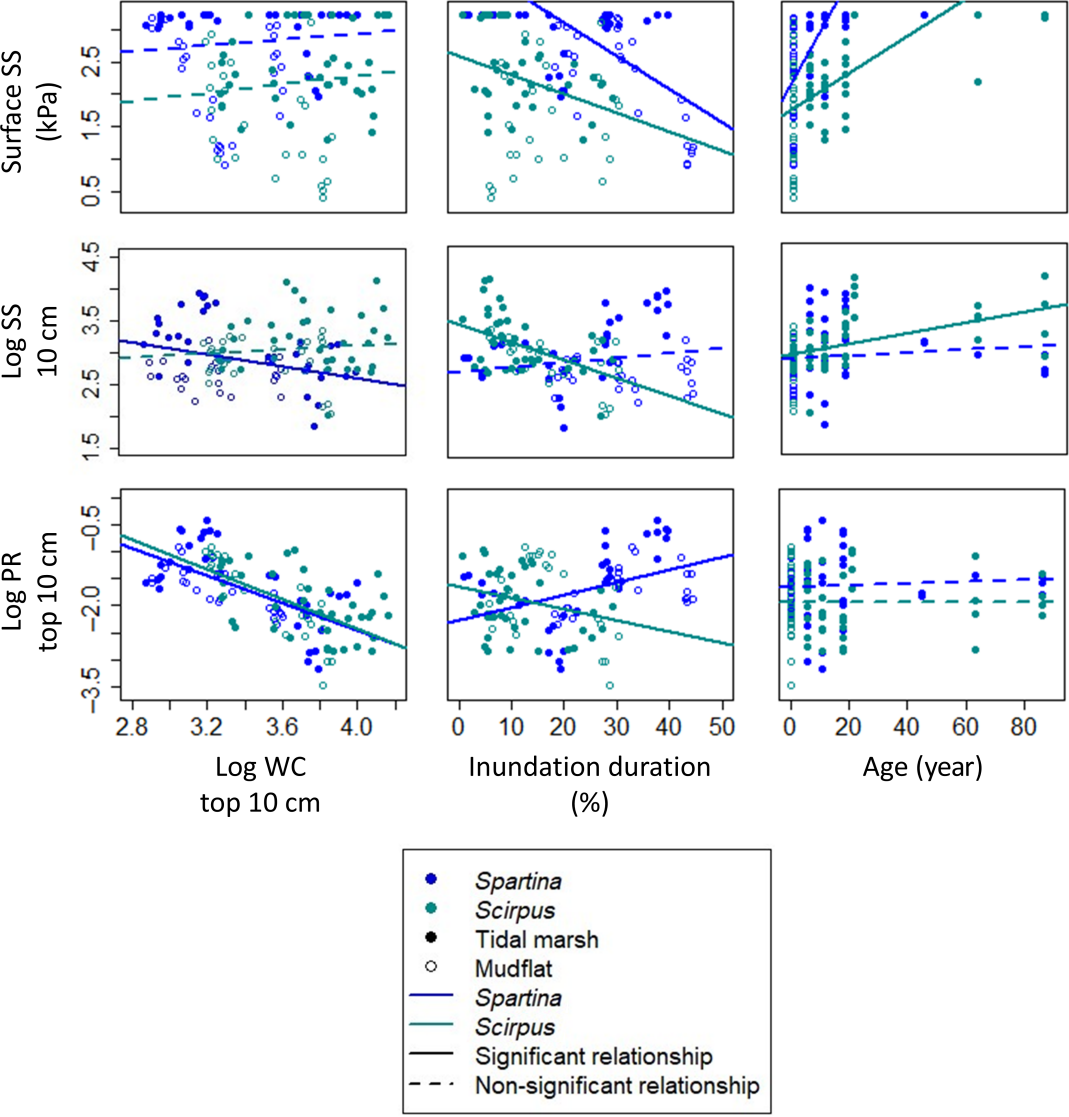
Relationships between surface shear strength (Surface SS), logarithm of shear strength at 10 cm depth (Log SS 10 cm), logarithm of average penetration resistance of the top 10 cm (Log PR top 10 cm), logarithm of sediment water content of 0–10 cm (WC top 10 cm), inundation duration, and age for all measured locations. Points represent an average of three replicates. Blue colors indicate measurements in the *Spartina*‐dominated marshes, green colors in the *Scirpus*‐dominated marshes. Closed points are measurements in the vegetated tidal marsh (high, mid, and pioneer combined), while open points are measurements at the vegetation edge and on the bare tidal flat. A continuous line indicates a significant censored (for surface shear strength) or linear (for shear strength at 10 cm and penetration resistance) regression (*p* < 0.05) between two variables, and a dashed line a nonsignificant regression.

Sediment water content and inundation duration had a negative relationship with some sediment stability measurements (Figure 5). In addition, relationships between sediment stability proxies and sediment water content, inundation duration, and age were species‐dependent (Figure 5). For both species, surface shear strength decreased significantly with inundation duration (*p* < 0.001 for *Spartina* dominated and *p* = 0.027 for *Scirpus* dominated) and increased significantly with age (*p* < 0.001 for both species). Shear strength at 10 cm decreased significantly with increasing sediment water content for the *Spartina*‐dominated marshes (*F*_1,55_ = 6, *R* ^2^ = 0.09, *p* = 0.014). On the other hand, shear strength at 10 cm decreased significantly with increasing inundation duration (*F*_1,63_ = 25, *R* ^2^ = 0.28, *p* < 0.001) and with increasing age (*F*_1,63_ = 17, *R* ^2^ = 0.20, *p* < 0.001) for the *Scirpus‐*dominated marshes. Both the *Spartina*‐ and *Scirpus*‐dominated mashes showed a significant negative relationship between average penetration resistance from 0 to 10 cm depth and sediment water content (*Spartina*: *F*_1,55_ = 40, *R* ^2^ = 0.41, *p* < 0.001; *Scirpus*: *F*_1,63_ = 42, *R* ^2^ = 0.39, *p* < 0.001). In addition, penetration resistance was positively correlated with inundation duration in the *Spartina*‐dominated marshes (*F*_1,56_ = 14, *R* ^2^ = 0.19, *p* < 0.001) and negatively in the *Scirpus*‐dominated marshes (*F*_1,63_ = 5, *R* ^2^ = 0.06, *p* = 0.024).

Some of the relationships that we found between sediment stability parameters and explanatory variables, or absence thereof, result from partitioning the data by dominant pioneer species and not by individual marsh. Although we did not find a relationship between shear strength at 10 cm depth and inundation duration for all *Spartina*‐dominated marshes combined (Figure 5), a significant negative relationship did exist between these variables at the *Spartina*‐dominated marshes of Paulinapolder and Hellegat separately (Paulinapolder: *F*_1,16_ = 12, *R* ^2^ = 0.39, *p* = 0.003; Hellegat: *F*_1,14_ = 56, *R* ^2^ = 0.79, *p* < 0.001; Appendix S4: Figure S1). On the other hand, the significant relationship we found for the *Scirpus*‐dominated marshes combined between shear strength at 10 cm depth and inundation duration (Figure 5) did not exist for the *Scirpus*‐dominated marsh of Paardenschor separately (*F*_1,15_ = 0.068, *R* ^2^ = −0.06, *p* = 0.780; Appendix S4: Figure S1).

## DISCUSSION

To be of use for nature‐based shoreline protection in the long term, tidal marshes need to efficiently attenuate waves, vertically accrete with sea level rise, and be erosion resistant. The latter was the focus of this study. Mature marsh sediment beds can generally withstand very high flow velocities (Marin‐Diaz et al., 2022; Schoutens et al., 2022). However, if the goal is to create new tidal marshes for shoreline protection, the time for a stable tidal marsh sediment bed to develop needs to be considered (Schoutens et al., 2022). Since it is still unknown how long this may take, we aimed to study the rate at which sediment stability builds up after the establishment of pioneer vegetation species and which factors affect sediment stability and its development rate. Our results showed that (1) pioneer species, (2) fine fraction and water content of the sediment, and (3) inundation duration are the main drivers of marsh sediment stability. The development time of a stable marsh sediment bed is modulated by the colonizing species; *Spartina* increases sediment stability within 6 years, while *Scirpus* needs more than 18 years to have such an effect. These factors and time frames should be considered in marsh restoration and creation projects to ensure the development of an erosion‐resistant sediment bed by the time that shoreline erosion protection services are required.

### The effect of pioneer species on the buildup of sediment stability

We found a difference between species in the effect of vegetation on sediment stability: *Spartina* generally increased sediment stability, while *Scirpus* did not. This difference between species can be explained by their different clonal expansion strategy. *Spartina* forms denser vegetation tussocks than *Scirpus* (Cao, Zhu, Herman, et al., 2021). The higher shoot density of *Spartina* is inextricably related to a denser root system (Feher & Hester, 2018), thus leading to a stronger effect of roots on sediment stability (Marin‐Diaz et al., 2022).

At Hoofdplaat, *Spartina* did not have the same stabilizing effect on sediment stability as it did in the other two *Spartina* marshes. This may be attributed to a difference in sediment composition. Sediment at the *Spartina*‐dominated marsh of Hoofdplaat had a higher water content, indicating that it was finer, than the sediment at the *Spartina*‐dominated marshes of Paulinapolder and Hellegat. This is in line with Feagin et al. (2009), Lo et al. (2017), Evans et al. (2022), and Schoutens et al. (2019), who also found a stronger effect of vegetation on sediment strength in relatively sandy sediments than in fine sediments. Fine sediments are already cohesive without vegetation due to the presence of clay and silt particles. Coarse sediments with a high sand content are non‐cohesive, and therefore, roots binding the sediment matrix are expected to lead to a stronger increase in sediment stability than in fine sediments (Evans et al., 2022). On the other hand, Marin‐Diaz et al. (2022) found that a silty pioneer marsh did reduce the chance of surface erosion compared with a silty tidal flat, while this was not the case for a sandy pioneer marsh and sandy tidal flat, with the two latter being highly erodible. However, they did not specifically look at the effect of different pioneer species, which might explain this discrepancy.

At all tidal marshes with a mature zone, sediment shear strength was generally higher in this mature zone than in the pioneer zone. We expect that this higher sediment stability results from climax instead of pioneer marsh vegetation, lower inundation duration, and older age, implying more time for sediment consolidation. However, since all these variables are correlated, it is difficult to identify the primary cause for the difference in sediment stability between the pioneer and mature zone.

### The effect of sediment properties and inundation duration on sediment stability

The strong negative relationship between water content and penetration resistance that we observed has been found in previous studies as well (Da Silva et al., 2016; Vaz et al., 2001; Weaich et al., 1992). Surprisingly, we found no relationships between water content and surface shear strength, and only between water content and shear strength at 10 cm for the *Spartina*‐dominated marshes. Locations with a high water content were shown in our study to have a high fine fraction content, which implies a high cohesion, and were therefore expected to have a high shear strength (Brooks et al., 2021; Feagin et al., 2009; Lo et al., 2017). On the other hand, very muddy sediment can lead to poor sediment drainage (Brooks et al., 2021; Crooks et al., 2002), leading to very high water contents and thus reducing sediment stability (Grabowski et al., 2011). The combined effect of cohesion and water content on sediment stability is likely the reason for a lack of many significant relationships in our study. Overall, this emphasizes the importance of good sediment pore water drainage for the creation and restoration of erosion‐resistant marsh sediment beds.

As expected, we generally found negative relationships between sediment stability and inundation duration. Locations with low inundation duration are typically located higher within the tidal frame and are drained better (Stagg & Mendelssohn, 2010). Sediment consolidation mainly occurs when sediment dries out (Dong et al., 2020) and thereby increases sediment strength. However, we did not find a significant relationship between inundation duration and shear strength at 10 cm for the *Spartina* dominated marshes, which is expected to result from the effect of vegetation and sediment characteristics on shear strength that might have been stronger in this case. Contrary to our hypothesis, there was a significant positive relationship between inundation duration and penetration resistance for *Spartina*. This is likely caused by the strong negative relationship between penetration resistance and water content. Locations in *Spartina* dominated marshes with a low inundation duration have a high water content, which leads to a low penetration resistance.

### The buildup of sediment stability over time

We expected that pioneer vegetation presence would lead to increasing sediment stability over time, due to root growth, organic matter accumulation, fine sediment trapping, and lowering sediment water content by plant uptake of pore water and evapotranspiration (Barciela‐Rial et al., 2023; Brooks et al., 2021; Gyssels et al., 2005; Lo et al., 2017; Vuik et al., 2016). When combining mature marsh, pioneer marsh, and tidal flat, marsh age correlates indeed positively with surface shear strength for both species and with shear strength at 10 cm for only the *Scirpus*‐dominated marshes. However, these relations are strongly affected by the higher sediment stability of the much older, mature marsh locations. The older, mature marsh locations also have a higher surface elevation than the pioneer marsh and tidal flat locations, which lowers inundation duration and increases drying of the sediment, which is also expected to be a factor affecting the increased sediment stability. *Spartina* did enhance sediment stability compared with the bare tidal flat, but no significantly higher shear strength was observed at the 2004 vegetation edge (18 years of vegetation presence) than at the 2016 vegetation edge (6 years of vegetation presence). For surface shear strength, this could result from the fact that under *Spartina* at Paulinapolder and Hoofdplaat, the maximum measurable value of the shear vane was often reached, and the surface shear strength values might therefore be an underestimation. Theoretically, these values could have been higher at the 2004 vegetation edge than at the 2016 edge. However, since we did not observe such a difference in the shear strength of deeper sediment layers, it is unlikely that the surface layer further stabilized beyond 6 years of colonization, assuming shear strength is not lost once it is gained. Since the main increase in sediment stability occurs between 0 and 6 years after *Spartina* establishment, we conclude that the presence of *Spartina* increases sediment stability within the first 6 years, after which little further increase in sediment stability takes place. This corresponds to the data presented by Watts et al. (2003), who also reported that after 6 years of regular tidal coverage by the sea, sediment stability of a marsh restoration site reached the level of stability of an older, natural marsh.

### Implications for tidal marsh restoration and creation

Although tidal marsh sediment beds are generally considered to be very erosion resistant (Marin‐Diaz et al., 2022; Möller et al., 2014; Schoutens et al., 2022; Spencer et al., 2016) and unvegetated tidal flat sediment beds tend to be more vulnerable to erosion (Evans et al., 2022; Marin‐Diaz et al., 2022), we show here that the pioneer zone does not necessarily have more erosion‐resistant sediment beds than the tidal flat yet. A pioneer tidal marsh with less densely growing *Scirpus* vegetation will not develop an erosion‐resistant sediment bed within 18 years. Although such a tidal marsh could efficiently attenuate waves and accrete sediments and biomass (Blum et al., 2021; Fagherazzi et al., 2012; Mudd et al., 2010; Vuik et al., 2016), it might be vulnerable to erosion. In the case of a densely growing *Spartina* marsh however, a strong marsh sediment bed can develop within 6 years, which can be used for nature‐based protection of shorelines. To ensure the development of erosion‐resistant sediment beds in marsh restoration and creation projects, we should consider (1) pioneer species, (2) fine fraction and water content of the sediment, and (3) inundation duration.

Firstly, pioneer vegetation does not always significantly increase marsh sediment strength compared with the tidal flat (Chirol et al., 2021; Evans et al., 2022), and we show here that this effect is species‐dependent. A more densely growing species, such as *Spartina*, was found to be more effective in increasing sediment stability than a species with lower shoot density, such as *Scirpus*. Chirol et al. (2021) found that from several pioneer salt marsh species, *Puccinellia* spp., which is a densely growing salt marsh species, created the strongest sediment beds. *Spartina* significantly increased sediment strength compared with the tidal flat within the first 6 years of establishment, which makes it desirable to have densely growing species such as *Spartina* in the pioneer zone if we want to quickly create tidal marshes for flood protection. However, due to the salinity gradient in the Western Scheldt estuary, *Spartina* is more abundant in the more seaward, higher salinity zone of the estuary. If our specific aim is to create highly erosion‐resistant marsh sediment beds for shoreline protection, this could be a reason to favor tidal marsh restoration or creation projects in the more saline zone of the estuary where the densely growing species *Spartina* naturally occurs. Another option is to actively plant densely growing species in the more brackish zone, which could be a useful method in areas without a natural supply of seeds and propagules or where conditions prevent natural seedling establishment (Hudson et al., 2021). However, planting species in a zone where they naturally do not occur is not always successful if species, site conditions, or planting technologies are inappropriate (Liu et al., 2024). The growth and survival chances of species that are planned to be planted at a certain location should therefore be studied before starting large‐scale marsh restoration projects. If growth and survival chances are expected to be small, a different species, location, or planting technology might need to be chosen for the fast development of erosion‐resistant marsh sediment beds for shoreline protection.

When considering vegetation species of marshes for nature‐based shoreline protection in the long term, the effects of different vegetation species on wave attenuation and vertical accretion should also be considered. Although *Spartina* grows denser than *Scirpus* (positive effect on wave attenuation and sediment capturing), its flexural stiffness and average plant height are smaller than that of *Scirpus* (negative effect on wave attenuation and sediment capturing; Baaij et al., 2021; Temmerman et al., 2023; Zhu, Yang, et al., 2020). More research is needed to gain insight into the resulting wave attenuation and sediment‐capturing capacities of both species. It might be that different species are best at optimizing different functions (e.g., sediment erosion resistance, wave attenuation, vertical accretion). If this is the case, a trade‐off needs to be made depending on the main goal of the site.

Secondly, high fine fraction contents of marsh sediment lead to poor drainage conditions and might result in high water contents (Brooks et al., 2021). These high water contents will lower the erosion resistance of the sediment (Grabowski et al., 2011). Therefore, it is crucial to assess the grain size distribution of sediment in estuaries where marsh restoration or creation projects are planned. Drainage will also improve by the presence of creeks (Garbutt et al., 2006), which can be artificially dug if necessary. However, if it is decided to create artificial creeks, it is important that the correct creek geometry is constructed to ensure sufficient sediment supply (Brunetta et al., 2019). Too many or too large drainage channels can undermine resilience to sea level rise.

Thirdly, a high inundation duration can lead to marsh sediment beds that are prone to erosion, since less consolidation will occur, and water content will generally be higher. Especially concerning marsh restoration or creation projects where previously embanked, low‐lying land is inundated again, it is important to consider the area's elevation. If inundation duration is initially high, this could delay the formation of a stable sediment bed in the short term. Sediment nourishment or the use of structures (e.g., groins or breakwaters) to accelerate sediment settling could be relevant measures to increase bed elevation and reduce inundation duration (Baptist et al., 2021; Vuik et al., 2019). However, in the case of sediment nourishments, care should be taken that the sediment is not too sandy, as this will lead to easily erodible marshes (Marin‐Diaz et al., 2022).

We showed that the development rate of tidal marsh sediment stability depends on vegetation species, fine fraction and water content of the sediment, and inundation duration and that a tidal marsh will become more stable over time when evolving from a pioneer to climax marsh. Therefore, in order for erosion‐resistant sediment beds to develop in future marsh restoration and creation projects, it should be the aim to create densely vegetated tidal marshes with well‐draining, cohesive sediment beds at relatively high elevation. Although development of erosion resistance takes time, our study demonstrated that in the case of densely growing *Spartina* marshes, increased sediment bed stability is already reached after 6 years. The ability of *Spartina anglica* marshes to increase sediment bed stability within 6 years, in combination with wave attenuation and sediment accretion, offers promising perspectives to implement marsh restoration projects as a nature‐based shoreline protection strategy that can start to deliver its protective service within a reasonable amount of time.

## CONFLICT OF INTEREST STATEMENT

The authors declare no conflicts of interest.

## Supporting information


Appendix S1:

Appendix S2:

Appendix S3:

Appendix S4:

## Data Availability

Data (Stoorvogel et al., 2024) are available in the 4TU.ResearchData repository at https://doi.org/10.4121/844133cd-f6f5-4d2b-ae22-3e19f124d0eb.
