# Supplementary material for: Plant species, inundation, and sediment grain size control the development of sediment stability in tidal marshes
Source: Ecol Appl. 2025 Jan 20;35(1):e3078. doi: 10.1002/eap.3078 (PMC11744737; doi:10.1002/eap.3078)
Supplement: Supplementary file 1 — Appendix S1: [file EAP-35-e3078-s004.pdf]

Appendix S1

Journal: Ecological Applications

Plant species, inundation, and sediment grain size control the development of sediment stability in tidal marshes

Marte M. Stoorvogel, Jaco C. de Smit, Lauren E. Wiesebron, Jim van Belzen, Johan van de Koppel, Stijn Temmerman, Tjeerd J. Bouma

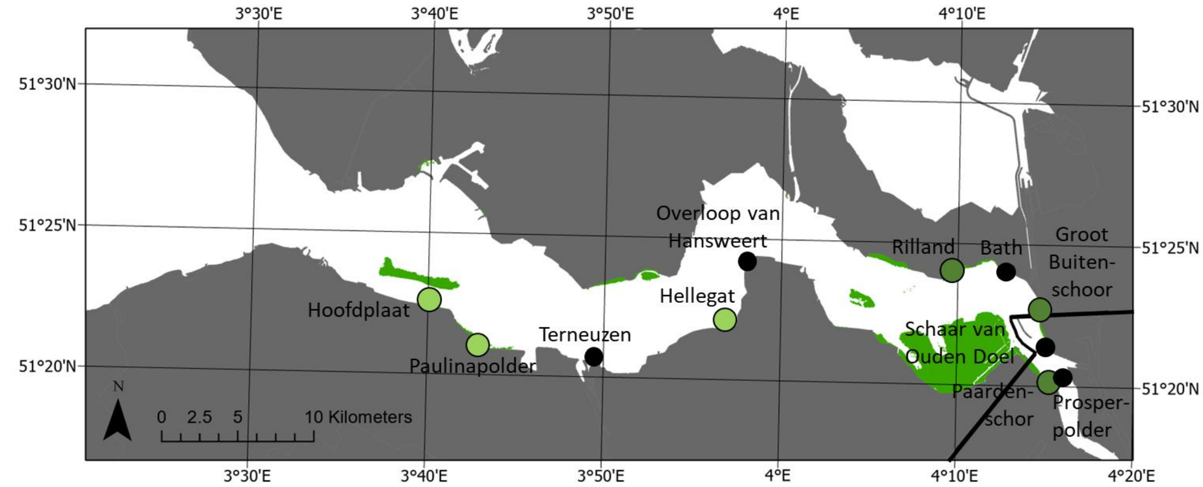

Figure S1 Map of the Western Scheldt estuary indicating the location of the studied tidal marshes (Hoofdplaat, Paulinapolder, Hellegat, Rilland, Groot Buitenschoor and Paardenschor), the salinity measurement stations (Terneuzen and Schaar van Ouden Doel), and the tide gauges (Terneuzen, Overloop van Hansweert, Bath, and Prosperpolder). The tidal marshes indicated with light green have *Spartina* as dominant pioneer species, the brackish marshes indicated with dark green have *Scirpus* as dominant pioneer species. The extent of tidal marshes in the Western Scheldt is shown in green and the black line is the Dutch-Belgian border.
