# Supplementary material for: Plant species, inundation, and sediment grain size control the development of sediment stability in tidal marshes
Source: Ecol Appl. 2025 Jan 20;35(1):e3078. doi: 10.1002/eap.3078 (PMC11744737; doi:10.1002/eap.3078)
Supplement: Supplementary file 2 — Appendix S2: [file EAP-35-e3078-s003.pdf]

Appendix S2

Journal: Ecological Applications

Plant species, inundation, and sediment grain size control the development of sediment stability in tidal marshes

Marte M. Stoorvogel, Jaco C. de Smit, Lauren E. Wiesebron, Jim van Belzen, Johan van de Koppel, Stijn Temmerman, Tjeerd J. Bouma

Table S1 Age, elevation, and inundation duration of the locations where we did our measurements along the transects in the six tidal marshes. TF stands for tidal flat and TM for tidal marsh.

Marsh	Transect	Location	Age (yr)	Elevation (m + NAP)	Inundation duration (%)
Paulinapolder	1	18 yr	18	1.31	27.72
	1	11 yr	11	1.30	27.72
	1	6 yr	6	1.15	30.38
	1	0 yr	0	1.00	32.83
	1	TF 1m	0	0.95	33.43
	1	TF 6m	0	0.92	33.89
	2	18 yr	18	1.30	27.90
	2	11 yr	11	1.25	28.58
	2	6 yr	6	1.29	28.08
	2	0 yr	0	1.16	30.06
	2	TF 1m	0	1.17	29.91
	2	TF 6m	0	1.14	30.38
	3	18 yr	18	1.33	27.21
	3	11 yr	11	1.28	28.08
	3	6 yr	6	1.25	28.58
	3	0 yr	0	1.14	30.38
	3	TF 1m	0	1.13	30.71
	3	TF 6m	0	1.12	30.88
Hoofdplaat	1	18 yr	18	2.02	12.50
	1	11 yr	11	1.83	16.92
	1	6 yr	6	1.80	17.82
	1	0 yr	0	1.75	18.74
	1	TF 1m	0	1.78	18.28
	1	TF 6m	0	1.71	19.60
	1	Mid TM	63	2.66	1.81
	1	High TM	86	2.45	4.26
	2	18 yr	18	1.84	16.92
	2	11 yr	11	1.70	19.82
	2	6 yr	6	1.74	18.94
	2	0 yr	0	1.67	20.48
	2	TF 1m	0	1.67	20.68
	2	TF 6m	0	1.62	21.51
	2	Mid TM	45	2.31	6.62
	2	High TM	86	2.44	4.40
	3	18 yr	18	2.07	11.44
	3	11 yr	11	1.72	19.38

**Marsh**	**Transect**	**Location**	**Age (yr)**	**Elevation (m + NAP)**	**Inundation duration (%)**
Hoofdplaat	3	6 yr	6	1.70	19.82
	3	0 yr	0	1.59	22.33
	3	TF 1m	0	1.61	21.92
	3	TF 6m	0	1.57	22.53
	3	Mid TM	45	2.23	8.16
	3	High TM	86	2.80	0.87
Hellegat	1	18 yr	18	0.81	35.71
	1	11 yr	11	0.68	37.68
	1	6 yr	6	0.55	39.70
	1	0 yr	0	0.40	42.26
	1	TF 1m	0	0.33	43.25
	1	TF 6m	0	0.33	43.25
	2	18 yr	18	0.68	37.68
	2	6 yr	6	0.58	39.40
	2	0 yr	0	0.35	43.08
	2	TF 1m	0	0.32	43.41
	2	TF 6m	0	0.27	44.43
	3	18 yr	18	0.69	37.68
	3	6 yr	6	0.58	39.24
	3	0 yr	0	0.34	43.25
	3	TF 1m	0	0.28	44.09
	3	TF 6m	0	0.28	44.26
Groot Buitenschoor	1	18 yr	18	2.22	15.09
	1	11 yr	11	1.68	23.58
	1	6 yr	6	1.46	26.89
	1	0 yr	0	1.45	27.17
	1	TF 1m	0	1.40	27.75
	1	TF 6m	0	1.35	28.50
	1	Mid TM	21	2.65	7.86
	1	High TM	21	2.85	4.98
	2	18 yr	18	1.80	21.93
	2	11 yr	11	1.55	25.53
	2	6 yr	6	1.57	25.23
	2	0 yr	0	1.55	25.53
	2	TF 1m	0	1.51	26.29
	2	TF 6m	0	1.46	27.03
	2	Mid TM	21	2.57	9.11
	2	High TM	21	2.89	4.59
	3	18 yr	18	1.86	20.84
	3	11 yr	11	1.44	27.17
	3	6 yr	6	1.31	29.08
	3	0 yr	0	1.31	29.08

Marsh	Transect	Location	Age (yr)	Elevation (m + NAP)	Inundation duration (%)
Groot Buitenschoor	3	TF 1m	0	1.26	29.83
	3	TF 6m	0	1.22	30.44
	3	Mid TM	63	2.66	7.86
	3	High TM	63	3.34	0.75
Paardenschor	1	11 yr	11	2.83	5.36
	1	6 yr	6	2.71	7.09
	1	0 yr	0	2.63	8.16
	1	TF 1m	0	2.61	8.64
	1	TF 6m	0	2.56	9.44
	2	11 yr	11	2.74	6.65
	2	6 yr	6	2.60	8.80
	2	0 yr	0	2.55	9.59
	2	TF 1m	0	2.50	10.38
	2	TF 6m	0	2.47	10.72
	2	Mid TM	18	2.82	5.36
	2	High TM	18	2.81	5.50
	3	11 yr	11	3.03	2.92
	3	6 yr	6	2.87	4.85
	3	0 yr	0	2.77	6.08
	3	TF 1m	0	2.79	5.79
	3	TF 6m	0	2.76	6.22
Rilland	1	18 yr	18	2.53	10.62
	1	11 yr	11	2.45	11.95
	1	6 yr	6	2.26	15.35
	1	0 yr	0	2.16	17.16
	1	TF 1m	0	2.13	17.68
	1	TF 6m	0	1.99	19.93
	1	Mid TM	63	2.84	5.55
	1	High TM	86	2.67	8.15
	2	18 yr	18	2.41	12.46
	2	11 yr	11	2.34	13.71
	2	6 yr	6	2.32	14.08
	2	0 yr	0	2.32	14.08
	2	TF 1m	0	2.26	15.35
	2	TF 6m	0	2.19	16.44
	2	Mid TM	63	2.59	9.61
	2	High TM	86	2.83	5.83
	3	18 yr	18	2.57	9.95
	3	11 yr	11	2.50	10.95
	3	6 yr	6	2.41	12.46
	3	0 yr	0	2.42	12.46
	3	TF 1m	0	2.36	13.35

**Marsh**	**Transect**	**Location**	**Age (yr)**	**Elevation (m + NAP)**	**Inundation duration (%)**
Rilland	3	TF 6m	0	2.26	15.17
	3	Mid TM	86	2.95	4.01
	3	High TM	86	2.66	8.31
