# Supplementary material for: Plant species, inundation, and sediment grain size control the development of sediment stability in tidal marshes
Source: Ecol Appl. 2025 Jan 20;35(1):e3078. doi: 10.1002/eap.3078 (PMC11744737; doi:10.1002/eap.3078)
Supplement: Supplementary file 3 — Appendix S3: [file EAP-35-e3078-s002.pdf]

Appendix S3

Journal: Ecological Applications

Plant species, inundation, and sediment grain size control the development of sediment stability in tidal marshes

Marte M. Stoorvogel, Jaco C. de Smit, Lauren E. Wiesebron, Jim van Belzen, Johan van de Koppel, Stijn Temmerman, Tjeerd J. Bouma

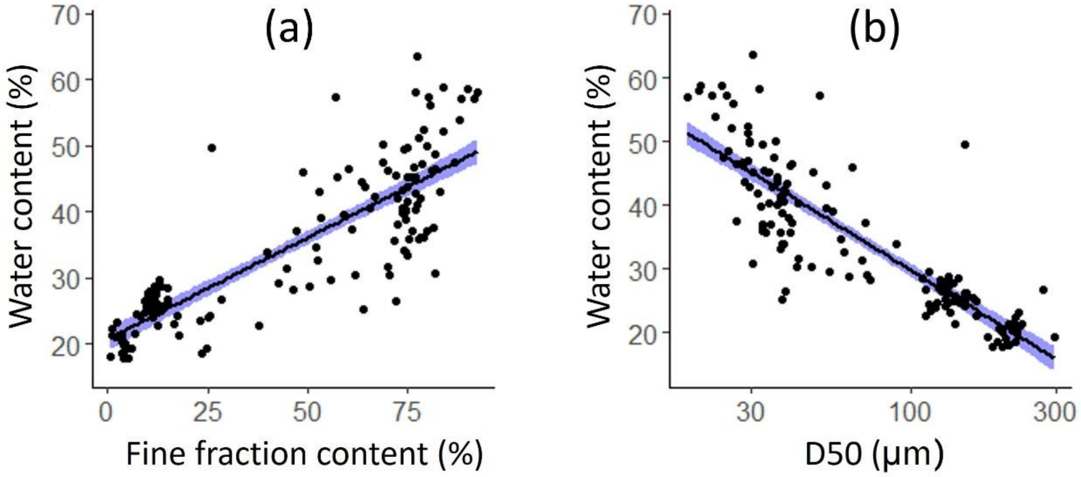

Figure S1 Relationships between water content and (a) fine fraction content and (b) D50, deduced from a selection of 149 sediment bed samples of all tidal marshes. The x axis in (b) is on a logarithmic scale. The 95% confidence interval bands are indicated in blue.
