# Supplementary material for: Plant species, inundation, and sediment grain size control the development of sediment stability in tidal marshes
Source: Ecol Appl. 2025 Jan 20;35(1):e3078. doi: 10.1002/eap.3078 (PMC11744737; doi:10.1002/eap.3078)
Supplement: Supplementary file 4 — Appendix S4: [file EAP-35-e3078-s001.pdf]

Appendix S4

Journal: Ecological Applications

Plant species, inundation, and sediment grain size control the development of sediment stability in tidal marshes

Marte M. Stoorvogel, Jaco C. de Smit, Lauren E. Wiesebron, Jim van Belzen, Johan van de Koppel, Stijn Temmerman, Tjeerd J. Bouma

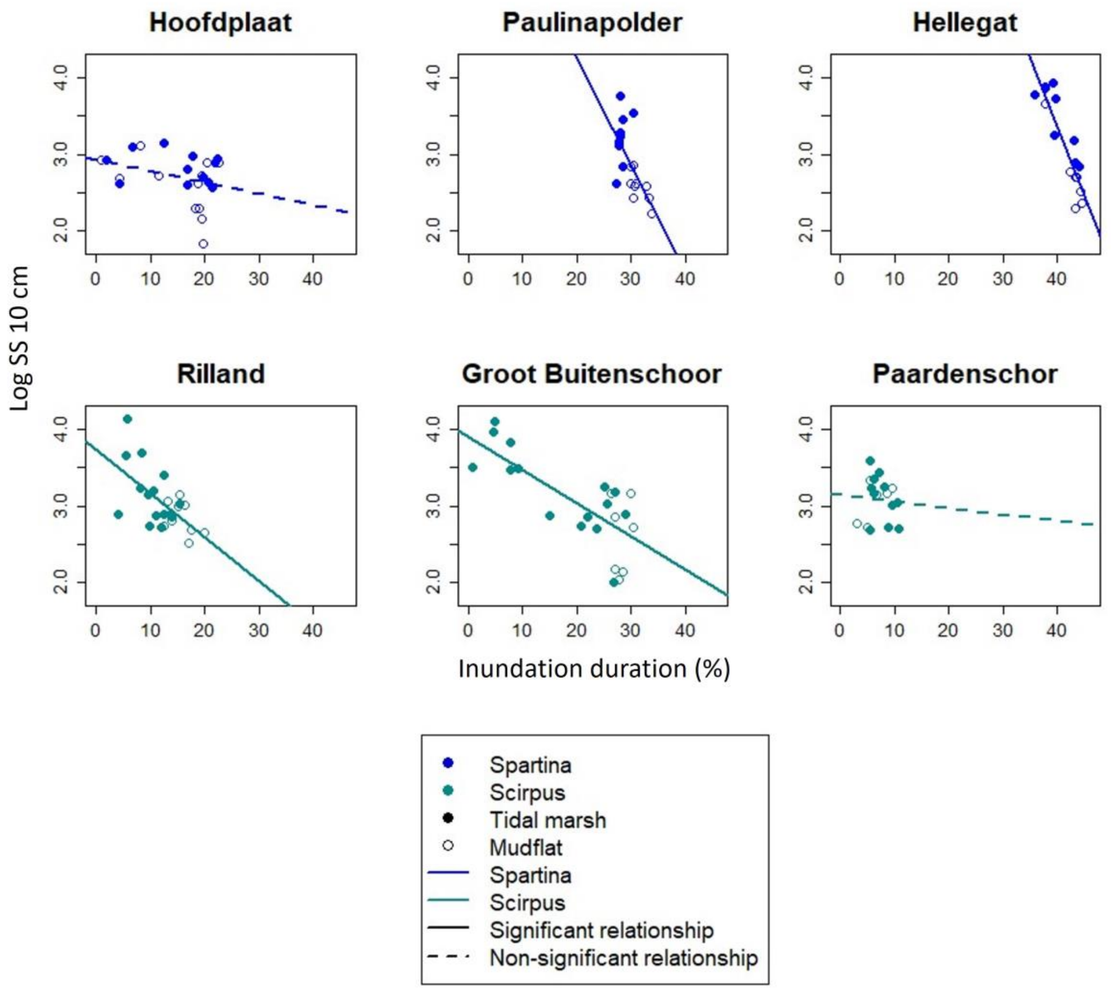

Figure S1 Relationships between the logarithm of shear strength at 10 cm depth (Log SS 10 cm) and inundation duration for all measured locations separated per tidal marsh. Points represent an average of three replicates. Blue colours indicate measurements in the *Spartina* marshes, green colours in the *Scirpus* marshes. Closed points are measurements in the vegetated tidal marsh (high, mid, and pioneer combined), while open points are measurements at the vegetation edge and on the bare tidal flat. A continuous line indicates a significant linear regression ($p < 0.05$), a dashed line a non-significant linear regression.
